# Antioxidant, Anti-Inflammatory, and Analgesic Activities of Aqueous Extract of *Diploknema butyracea* (Roxb.) H.J. Lam Bark

**DOI:** 10.1155/2020/6141847

**Published:** 2020-12-02

**Authors:** Sumit Bahadur Baruwal Chhetri, Deepa Khatri, Kalpana Parajuli

**Affiliations:** Department of Pharmaceutical Sciences, School of Health and Allied Sciences, Pokhara University, Pokhara 33700, Nepal

## Abstract

*Diploknema butyracea* (Roxb.) H.J. Lam is a multipurpose tree used by the Nepalese indigenous people for medicinal purposes such as rheumatism, asthma, and ulcer and other purposes such as cooking and lighting. However, there is no scientific evidence for the medicinal uses of this plant. The present study aimed to explore the phytochemical constituents, estimate the total phenolic content, evaluate antioxidant activity, and investigate the *in vivo* anti-inflammatory and analgesic activities of aqueous extract of *Diploknema butyracea* (Roxb.) H.J. Lam bark (ADBB). Phytochemical screening was performed using standard methods. The total phenolic content was determined using the Folin–Ciocalteu method. The *in vitro* antioxidant activity was determined using 2, 2-diphenyl-1-picrylhydrazyl radical scavenging assay and nitric oxide radical scavenging assay. For the *in vivo* studies, the plant extract was given in three different doses (50, 100, and 200 mg/kg body weight) to male albino Wistar rats. Anti-inflammatory and analgesic studies were carried out using the carrageenan-induced rat paw edema and the hot plate method, respectively. Results revealed the presence of different phytoconstituents such as flavonoids, tannins, glycosides, terpenoids, and carbohydrates together with a considerable amount of phenolic compounds. Antioxidant assays indicated the potent antioxidant activity of the plant extracts. The higher dose of *D. butyracea* (200 mg/kg) exhibited a maximum and significant inhibition (53.20%) of rat hind paw edema volume at 4 h and showed a greater increment in latency time (12.15 ± 1.81 sec) in the hot plate test at 120 min. The present study demonstrated the antioxidant, anti-inflammatory, and analgesic potential of ADBB, which supports its traditional medicinal use.

## 1. Introduction

Inflammation is a crucial part of the host defense mechanism in the human body, which helps to remove harmful stimuli such as pathogens and toxicants and restore the damaged tissue [1]. The inflammation can be categorized as acute and chronic inflammation. Acute inflammation is a short-duration inflammatory condition characterized by redness, swelling, heat, pain, and loss of tissue function. Chronic inflammation is a prolonged inflammatory condition characterized by concurrent tissue destruction and repairment with fibrosis [2]. It leads to several chronic diseases including cancer, arthritis, obesity, diabetes, cardiovascular diseases, and neurological diseases [3].

Pain is a displeasing sensation, which often shows the indication of various health-related disorders and injuries in the human body [4]. It interferes with a person's physical and mental capabilities and thus, has a negative impact on the quality of life [5].

Nonsteroidal anti-inflammatory drugs (NSAIDs) are effective in treating a variety of pain and inflammatory conditions. However, the long-term use of NSAIDs is becoming highly controversial due to their association with serious side effects such as gastrointestinal ulcers, hemorrhage, and renal damage. In comparison to these synthetic drugs, herbal medicines are in greater demand because of their affordability, accessibility, and safety profile. Therefore, nowadays, people are more focused on the use of ethnobotanical knowledge to discover herbal medicine [6].

*Diploknema butyracea* (Roxb.) H.J. Lam (synonyms: *Aesandra butyracea* (Roxb.) Baehni, *Madhuca butyracea* (Roxb.) J.F. Macbr., *Illipe butyracea* (Roxb.) Engl., *Mixandra butyracea* (Roxb.) Pierre ex L. Planch., *Vidoricum butyraceum* (Roxb.) Kuntze and *Bassia butyracea *Roxb.; family: Sapotaceae; Nepali name: Chiuri; English name: Indian-butter nut; Hindi name: Phalwara; Chinese name: Zang lan) is a deciduous tree about 20 m tall, growing in the sub-Himalayan range at an altitude of 300 to 1500 m. It is widely used among tribal communities of Nepal, India, Tibet, and Bhutan. *Chepang*, an indigenous Tibeto-Burman ethnic group of Nepal, uses various parts of *D. butyracea* for medicinal and other purposes. Fruits are used as dietary supplements, whereas seed fats are used for various purposes such as cooking, lighting lamps, and manufacturing soap, candles, and hair oils [7, 8]. Seed fat is also used as an ointment in rheumatism, boils, pimples, and burn, and as an emollient for chapped hands and feet in winter. The bark of the tree is used in the treatment of asthma, indigestion, ulcers, itching, hemorrhage, contraction of limbs, wounds, rheumatic pain, inflammation of the tonsils, leprosy, and diabetes [8–10].

*D. butyracea* has been used for various medicinal purposes by different tribal groups of Nepal since the ancient period, but there is limited scientific exploration. To our knowledge, there are no previous scientific studies on the antioxidant, anti-inflammatory, and analgesic activities of *D. butyracea*. Therefore, to explore the medicinal uses of this plant scientifically, our study focused to evaluate the antioxidant, anti-inflammatory, and analgesic activities of aqueous extract of *D. butyracea* bark (ADBB) of Nepal. Furthermore, the phytochemical constituents and total phenolic content of ADBB were assessed, which help to support these biological activities.

## 2. Materials and Methods

### 2.1. Chemicals

Carrageenan was obtained from HiMedia Laboratories Pvt. Ltd., India. Folin–Ciocalteu reagent and ascorbic acid were purchased from Sigma-Aldrich, USA. DPPH (2, 2-diphenyl-1-picrylhydrazyl) and gallic acid were purchased from Wako Pure Chemical Industries Ltd., Osaka, Japan. All other chemicals used were of analytical grade.

### 2.2. Plant Material

The bark of *D. butyracea* was collected from Pokhara, Kaski district, Nepal, in September 2016 and was identified and authenticated by botanist Dr. Radheshyam Kayastha. The voucher specimen (PUCD–2017–01) was deposited at the Laboratory of Pharmacognosy, School of Health and Allied Sciences, Pokhara University, Pokhara, Nepal.

### 2.3. Extraction and Yield Percent Calculation

The dried bark of *D. butyracea* (25 g) was macerated twice with distilled water in the sample to solvent ratio 1 : 7 (w/v) for 48 h at room temperature. Then, the extract obtained was filtered and concentrated under reduced pressure in a rotary evaporator (Büchi Rotavapor, Germany) to obtain the dried extract for further study. The percentage of extract yield was calculated by using the following formula:

(1) $$\begin{array}{c} \text{Extract yield}\left(\%\right)=\frac{\text{Weight of dried extract}}{\text{Weight of dried bark}}\times 100. \end{array}$$

### 2.4. Animals and Experimental Design

Male albino Wistar rats (150–180 g) were purchased from The Science House, Pokhara, Nepal. Rats were randomly divided into five groups, each containing six rats (Table 1). Each group of rats was housed individually in polypropylene cages under controlled environment conditions (25 ± 3°C temperature, 55 ± 5% humidity, and 12 : 12 h light-dark cycle). Rats were acclimatized in the laboratory of Pokhara University for 3 weeks prior to the study and fed with food and water *ad libitum.* All rats were fasted for 14 h before the conduction of the experiment.

### 2.5. Ethical Statement

Ethical clearance was obtained from the Institutional Review Committee (IRC) of Pokhara University (Reference number: 130-073-074), and all activities were conducted according to Ethical Guidelines for the Care and Use of Animals in Health Research in Nepal.

### 2.6. Phytochemical Screening

Preliminary phytochemical screening of the plant extract was performed as described previously [11, 12] to detect the presence of phytoconstituents such as alkaloids, carbohydrates, flavonoids, glycosides, saponins, tannins, terpenoids, proteins, and amino acids in ADBB.

### 2.7. Determination of Total Phenolic Content

The total phenolic content in ADBB was determined using the Folin–Ciocalteu method as described previously [11]. Gallic acid was used as a standard in the determination of total phenolic content. The quantitative estimation was performed with the help of the equation (*y* = 0.013*x* – 0.164) obtained from the standard curve. The total phenolic content was expressed as mg gallic acid equivalent (GAE) per gram dry extract weight.

### 2.8. Antioxidant Activity

#### 2.8.1. DPPH Radical Scavenging Assay

The DPPH radical scavenging assay was carried out according to the method described earlier [13] with slight modification. Ascorbic acid was taken as standard. In brief, an equal volume of the extract/standard (1 *μ*g/mL, 10 *μ*g/mL, and 100 *μ*g/mL) and 60 *μ*M DPPH solution were mixed in a test tube. The reaction mixture was kept at room temperature in the dark for 30 min. The absorbance of the solution was measured at 517 nm using a UV-VIS spectrophotometer (Shimadzu UV-1800).

#### 2.8.2. Nitric Oxide Radical Scavenging Assay

Nitric oxide radical scavenging activity of plant extracts was measured using the Griess reagent [14]. This assay is based on the principle that decomposition of sodium nitroprusside (SNP) in an aqueous solution at physiological pH (7.2) results in the production of nitric oxide, which under aerobic conditions interacts with oxygen to form nitrate and nitrite ions that can be quantitatively estimated using Griess reagent. Curcumin was taken as standard. Briefly, 1 mL of extract/standard (1 *μ*g/mL, 10 *μ*g/mL, and 100 *μ*g/mL) was mixed with 1 mL of 5 mM sodium nitroprusside solution in standard phosphate buffer (pH 7.4) and incubated for 2.5 h at 29°C. After incubation, 2 mL of Griess reagent (sulphanilamide 1%, orthophosphoric acid 5%, and N-(1-Naphthyl)ethylenediamine dihydrochloride 0.1%) was added to the reaction mixture, and the absorbance was measured at 546 nm using a UV-VIS spectrophotometer.

(2) $$\begin{array}{c} \text{Antioxidant activity}\left(\%\right)=\frac{\text{Absorbance of control}-\text{Absorbance of sample}}{\text{Absorbance of control}}\times 100. \end{array}$$

The results were expressed in the terms of IC_50_ (concentration of the sample required for 50% inhibition), which was calculated from the graph of percentage inhibition versus concentration. All experiments were performed in triplicate.

### 2.9. Acute Toxicity Test

An acute toxicity test was carried out by following the Organization for Economic Co-operation and Development (OECD) guideline 425 for the testing of chemicals for acute oral toxicity [15]. Rats were divided into four groups, each consisting of 6 rats. The first group was considered a control group and received distilled water. The remaining three groups were orally treated with 500, 1000, and 2000 mg/kg ADBB, respectively. All rats were continuously monitored for any abnormal behavior during the first 4 h, and the number of deaths of rats was counted after 24 h.

### 2.10. Anti-Inflammatory Activity

The anti-inflammatory activity of the plant extract was evaluated using the carrageenan-induced rat paw edema model as described by Winter et al. [16] with slight modifications. Wistar rats of different groups were treated orally with standard drug diclofenac and plant extract, as mentioned in Table 1. After 60 min of drug/extract administration, edema was induced in the right hind paw of each rat by subplantar injection of 0.1 mL of freshly prepared carrageenan (1% (w/v) in normal saline). The volume of the right hind paw was measured by using a plethysmometer (UGO Basile 7140, Italy) before the injection and at 1, 2, 3, and 4 h of injection. The differences between the initial and final paw volume readings gave the measurement of inflammation index (*Ii*). The percentage inhibition of edema was calculated according to the following formula:

(3) $$\begin{array}{c} \%\text{ Edema inhibition}=\frac{\text{Control group }Ii-\text{Test group }Ii}{\text{Control group }Ii}\times 100. \end{array}$$

### 2.11. Analgesic Activity

The analgesic activity of plant extract was determined using the hot plate method as described previously by Khatri et al. [17], and the method description partly reproduces their wording. Wistar rats of different groups were treated orally with standard drug diclofenac and plant extract, as mentioned in Table 1. Rats were placed on the hot plate which was maintained at 55 ± 1°C. The latency time or reaction time was recorded before the administration of extract/standard drug (0 min) and after 30 min, 60 min, 120 min, and 180 min of extract/standard drug administration.

### 2.12. Statistical Analysis

All data are expressed as mean ± standard deviation (SD). The statistical analysis was carried out using Statistical Package for the Social Sciences (SPSS) 16.0 for Windows. All *in vivo* data were analyzed using the one-way ANOVA, followed by Dunnett's post hoc test. The *P* value of less than 0.05 was considered statistically significant.

## 3. Results and Discussion

### 3.1. Extract Yield Percent

The percentage of aqueous extract yield was found to be 26.5%.

### 3.2. Phytochemical Screening

The exploration of bioactive and disease-mitigating chemical constituents from traditional medicinal plants has been gaining a tremendous amount of attention in the field of drug discovery. The qualitative analysis of the ADBB revealed the presence of phytochemical constituents such as flavonoids, tannins, glycosides, terpenoids, and carbohydrates. Awasthi and Mitra [18] also reported the presence of chemical constituents such as triterpenoids (*α*-amyrin acetate, *β*-amyrin acetate, and friedelin), steroid (*α*-spinasterol), steroid glycoside (*β*-D-glucoside of *β*-sitosterol), and 3*β*-palmitoxy-olea-12en-28-ol from the bark of *D. butyracea*. These phytoconstituents are known to possess useful biological activities such as anticancer, antioxidant, anti-inflammatory, analgesic, and wound-healing actions and, as a result, have led to enormous attention in pharmaceutical industries [19, 20].

### 3.3. Total Phenolic Content

Phenolic compounds are the pronounced secondary metabolite of the plant, which act as antiaging, antimicrobial, anticancer, anti-inflammatory, antioxidant, antiproliferative, cardioprotective, and immune system-promoting agents [21, 22]. The total phenolic content of ADBB was found to be 228.53 ± 0.65 mg GAE/g dry extract weight, which may significantly contribute to the various biological activities and became fruitful in the promotion of human health.

### 3.4. Antioxidant Assay

#### 3.4.1. DPPH Radical Scavenging Assay

The hydrogen atom or electron-donating potential of the plant extract was measured from the bleaching of the deep violet color of DPPH solution. The greater the bleaching of deep violet color of DPPH solution, the greater will be the antioxidant activity. Our results showed that ADBB and ascorbic acid exhibit the capacity to reduce the DPPH free radical with an IC_50_ value of 8.43 *μ*g/mL and 2.28 *μ*g/mL, respectively.

#### 3.4.2. Nitric Oxide Radical Scavenging Assay

In the present study, ADBB was effectively able to compete with oxygen which results in a decrease in the production of nitrate and nitrite ions. The nitric oxide radical scavenging activity of ADBB was found to be less (IC_50_ value = 148.33 *μ*g/mL) than that of the standard curcumin (IC_50_ value = 102.99 *μ*g/mL).

The mechanism behind these free radical scavenging activities of ADBB may be attributed to the presence of phenolic compounds, which have been reported to be associated with antioxidative action in living organisms as it acts as a hydrogen donor, reducing agents, and scavenger of singlet oxygen and free radicals [23, 24].

### 3.5. Acute Toxicity Test

ADBB was found to be safe in all three tested doses. No death and toxicity signs were reported throughout the study period. In accordance with this toxicity test, three treatment doses of 50, 100, and 200 mg/kg body weight were selected for further *in vivo* studies.

### 3.6. Anti-Inflammatory Activity

The carrageenan-induced rat paw edema model is a suitable test for evaluating anti-inflammatory activity. Carrageenan is a high-molecular weight linear polysaccharide that releases inflammatory and proinflammatory mediators such as prostaglandins, leukotrienes, histamine, and bradykinin [25]. The inflammation induced by carrageenan consists of two phases. In the first phase of inflammation (0-1 h), inflammatory mediators such as histamine, bradykinin, and serotonin are released, while in the second phase of inflammation (1–6 h), biologically active inflammatory mediators such as prostaglandins and various cytokines such as IL-1*β*, IL-6, IL-10, and TNF-*α* are released [26]. In the present study, ADBB showed an inhibitory effect on rat hind paw edema in both phases of inflammation with a pronounced inhibitory effect in the second phase, which suggests that ADBB may have inhibited the inflammatory mediators that are released in various phases of inflammation. The anti-inflammatory activity of ADBB and the standard drug diclofenac is summarized in Table 2. The extracts displayed significant dose-dependent inhibition of carrageenan-induced paw edema. The ADBB at a dose of 200 mg/kg shows a similar effect to that of the standard drug diclofenac.

The exact mechanism behind the anti-inflammatory activity of *D. butyracea* is not known. However, the presence of various phytochemical constituents such as flavonoids, tannins, terpenoids, and glycosides in ADBB may have contributed to its anti-inflammatory action. Many studies have reported the anti-inflammatory role of flavonoids. Flavonoids can inhibit the expression of isoforms of inducible nitric oxide synthase, cyclooxygenase, and lipoxygenase, which are responsible for the production of various inflammatory mediators [27]. They also inhibit the regulatory enzyme protein kinase by competitively binding to ATP at catalytic sites on the enzymes and show the inhibition of the inflammatory response [28]. Tannins are also reported to show anti-inflammatory activity by inhibiting the cyclooxygenase (COX-1) enzyme [29]. Terpenoids are reported to possess anti-inflammatory activity by inhibiting the expression of tumor necrosis factor (TNF- *α*), prostaglandin synthesis, COX enzymes, inducible nitric oxide synthase enzymes, and cytokines such as IL-2, IL-4, and IL-6 [30]. *α*-Amyrin acetate, which is also present in the bark of *D. butyracea* [18], has been reported to possess an anti-inflammatory property by inhibiting the activity of COX-2 enzyme [31]. Similarly, *β*-sitosterol-*β*-D-glucoside has been also reported to exhibit anti-inflammatory activity in lipopolysaccharide-stimulated RAW 264.7 murine macrophages by reducing the production of nitric oxide, tumor necrosis factor-alpha (TNF-*α*), and interleukin 1 beta (IL-1*β*) [20]. Therefore, the anti-inflammatory effect of ADBB could be due to the presence of these phytoconstituents.

Nitric oxide plays an important role in inflammation. It is a key mediator in various phases of carrageenan-induced rat paw inflammation. It stimulates cyclooxygenase activity, which results in the overproduction of proinflammatory prostaglandins. It also reacts with superoxide anion to form the potent oxidant peroxynitrite, which plays a critical role in the inflammation process [32, 33]. In the present study, ADBB showed an inhibition of nitric oxide radical, which supports the anti-inflammatory role of ADBB.

### 3.7. Analgesic Activity

The analgesic activity of ADBB was assessed using the hot plate method, which is a thermal-induced pain state model. This method is useful for demonstrating the centrally mediated analgesic activity, which predominantly focuses on changes above the spinal cord level [34]. The result of the analgesic activity of the standard drug and plant extract is presented in Table 3. There was an increase in reaction time at all time points compared to baseline values within the same treatment groups. A dose-dependent increment in reaction time was observed, and the highest reaction time was noticed for *D. butyracea* (200 mg/kg), which was quite comparable to that of the standard drug diclofenac. This finding suggests that ADBB possesses centrally mediated antinociceptive activity. The analgesic activity of ADBB may be attributed to the presence of phytoconstituents such as tannins, terpenoids, glycosides, flavonoids, and phenolic compounds. Several phytoconstituents such as tannins, flavonoids, and triterpenoids were reported to possess analgesic properties [35, 36]. Further studies at the molecular level will provide the detailed mechanism behind the analgesic activity of ADBB.

## 4. Conclusions

The present study on *D. butyracea* shows potent antioxidant activity and significant anti-inflammatory and analgesic activities which support the traditional use of *D. butyracea* against various pain and inflammatory conditions. The reasons behind these activities of plant extracts might be due to the presence of various bioactive chemical constituents. Hence, further investigation should be carried out to determine the exact mechanism behind anti-inflammatory and analgesic activities and to isolate the active constituents responsible for these pharmacological activities.

## Figures and Tables

**Table 1:** Treatment protocols in different groups of rats for anti-inflammatory and analgesic activities.

Groups (*n* = 6)	Treatment protocols
Group I (control group)	Distilled water
Group II (standard group)	Diclofenac (50 mg/kg body weight)
Group III	ADBB (50 mg/kg body weight)
Group IV	ADBB (100 mg/kg body weight)
Group V	ADBB (200 mg/kg body weight)

*Note.* ADBB: aqueous extract of *D. butyracea* bark.

**Table 2:** Effect of aqueous extract of *D. butyracea* bark on carrageenan-induced rat paw edema.

Groups (*n* = 6)	Inflammation index in mL (% inhibition of paw edema volume)			
	1 h	2 h	3 h	4 h
Control	0.213 ± 0.07	0.343 ± 0.05	0.468 ± 0.04	0.406 ± 0.05
Diclofenac 50 mg/kg	0.143 ± 0.02^*∗*^ (32.86%)	0.203 ± 0.01^*∗*^ (40.81%)	0.250 ± 0.01^*∗*^ (46.58%)	0.173 ± 0.01^*∗*^ (57.38%)
ADBB 50 mg/kg	0.165 ± 0.03 (22.53%)	0.235 ± 0.03^*∗*^ (31.48%)	0.295 ± 0.01^*∗*^ (36.96%)	0.223 ± 0.04^*∗*^ (45.07%)
ADBB 100 mg/kg	0.156 ± 0.03 (26.76%)	0.216 ± 0.02^*∗*^ (37.02%)	0.260 ± 0.03^*∗*^ (44.44%)	0.203 ± 0.02^*∗*^ (50%)
ADBB 200 mg/kg	0.145 ± 0.03^*∗*^ (31.92%)	0.213 ± 0.005^*∗*^ (37.90%)	0.256 ± 0.01^*∗*^ (45.29%)	0.190 ± 0.03^*∗*^ (53.20%)

*Notes*. Data are expressed as mean ± standard deviation (*n* = 6), ^*∗*^*P* < 0.05, when compared to the control group, ADBB: aqueous extract of *D. butyracea* bark.

**Table 3:** Effect of aqueous extract of *D. butyracea* bark on hot plate-induced pain in rats.

Groups (*n* = 6)	Reaction time in seconds				
	0 min	30 min	60 min	120 min	180 min
Control	3.44 ± 0.66	3.80 ± 1.41	3.59 ± 0.53	3.86 ± 0.55	3.78 ± 0.49
Diclofenac 50 mg/kg	3.28 ± 0.46	7.28 ± 0.79^*∗*^	10.44 ± 0.72^*∗*^	12.49 ± 0.81^*∗*^	12.05 ± 1.98^*∗*^
ADBB 50 mg/kg	3.43 ± 0.63	4.25 ± 0.47	5.40 ± 0.57^*∗*^	6.22 ± 0.66^*∗*^	5.54 ± 0.55^*∗*^
ADBB 100 mg/kg	3.83 ± 0.54	5.03 ± 0.75	8.16 ± 1.30^*∗*^	10.24 ± 0.58^*∗*^	9.52 ± 0.89^*∗*^
ADBB 200 mg/kg	3.91 ± 0.46	5.58 ± 0.65^*∗*^	9.71 ± 1.01^*∗*^	12.15 ± 1.81^*∗*^	11.75 ± 1.02^*∗*^

*Notes*. Data are expressed as mean ± standard deviation (*n* = 6), ^*∗*^*P* < 0.05 when compared to the control group, ADBB: aqueous extract of *D. butyracea* bark.

## Data Availability

The data used to support the findings of this study are available from the corresponding author upon request.
